# The Gammaretroviral p12 protein has multiple domains that function during the early stages of replication

**DOI:** 10.1186/1742-4690-9-83

**Published:** 2012-10-04

**Authors:** Darren J Wight, Virginie C Boucherit, Mirella Nader, David J Allen, Ian A Taylor, Kate N Bishop

**Affiliations:** 1 Division of Virology, MRC National Institute for Medical Research, The Ridgeway, Mill Hill, London NW7 1AA, UK; 2 Division of Molecular Structure, MRC National Institute for Medical Research, The Ridgeway, Mill Hill, London, NW7 1AA, UK; 3 Current address: Virus Reference Department, Microbiology Services - Colindale, Health Protection Agency, 61 Colindale Avenue, London, NW9 5EQ, UK

**Keywords:** Retrovirus, MLV, p12, Post-entry events, Chromatin binding

## Abstract

**Background:**

The Moloney murine leukaemia virus (Mo-MLV) *gag* gene encodes three main structural proteins, matrix, capsid and nucleocapsid and a protein called p12. In addition to its role during the late stages of infection, p12 has an essential, but undefined, function during early post-entry events. As these stages of retroviral infection remain poorly understood, we set out to investigate the function of p12.

**Results:**

Examination of the infectivity of Mo-MLV virus-like particles containing a mixture of wild type and mutant p12 revealed that the N- and C-terminal regions of p12 are sequentially acting domains, both required for p12 function, and that the N-terminal activity precedes the C-terminal activity in the viral life cycle. By creating a panel of p12 mutants in other gammaretroviruses, we showed that these domains are conserved in this retroviral genus. We also undertook a detailed mutational analysis of each domain, identifying residues essential for function. These data show that different regions of the N-terminal domain are necessary for infectivity in different gammaretroviruses, in stark contrast to the C-terminal domain where the same region is essential for all viruses. Moreover, chimeras between the p12 proteins of Mo-MLV and gibbon ape leukaemia virus revealed that the C-terminal domains are interchangeable whereas the N-terminal domains are not. Finally, we identified potential functions for each domain. We observed that particles with defects in the N-terminus of p12 were unable to abrogate restriction factors, implying that their cores were impaired. We further showed that defects in the C-terminal domain of p12 could be overcome by introducing a chromatin binding motif into the protein.

**Conclusions:**

Based on these data, we propose a model for p12 function where the N-terminus of p12 interacts with, and stabilizes, the viral core, allowing the C-terminus of p12 to tether the preintegration complex to host chromatin during mitosis, facilitating integration.

## Background

Two hallmarks of retroviral replication are reverse transcription of the RNA genome into DNA and integration of this viral cDNA into the host cell chromatin. Whilst these enzymatic processes are well characterized, less is known about other early replication steps, for example, uncoating, cytoplasmic trafficking, nuclear entry and chromatin targeting. The timing of each step and the contribution of cellular factors in these processes are also poorly defined [1]. Several restriction factors inhibit various early events [2], and a better understanding of these stages of retroviral replication would help elucidate the mechanisms of restriction and aid the development of novel antiviral therapies for HIV/AIDS, as well as enhancing the design of retroviral gene therapy vectors.

All retroviruses encode a Gag polyprotein that is cleaved into individual proteins by the viral protease during maturation. In addition to the three main structural proteins, matrix (MA), capsid (CA) and nucleocapsid (NC), most retroviral Gag proteins contain additional cleavage products, several with unknown function(s). In many retroviral genera, the additional protein(s) is situated between MA and CA in the Gag polyprotein [3]. One such protein is p12 from murine leukaemia virus (MLV). The p12 protein carries a PPPY motif known as a late-domain (L-domain) required by all retroviruses to recruit the cellular ESCRT machinery needed for efficient viral budding [4]. Although HIV-1 does not code for a protein between MA and CA, it does contain an L-domain in the p6 protein located at the C-terminus of Gag [5,6]. As well as a role during the late stages of replication, p12 has an essential, but undefined, function during the early stages of replication [7].

It has been shown that replacing stretches of residues in the N- and C-terminus of p12 with alanines inhibits MLV replication before formation of the provirus [8]. Additionally, these regions are intolerant to insertional mutagenesis [9]. These p12 substitution mutant viruses exhibit two phenotypes with regard to the stage of arrest: 1) before/during reverse transcription and 2) post-reverse transcription but pre-integration of the viral cDNA [8]. Biochemical analysis of one p12 mutant with the second phenotype (PM14) revealed that its preintegration complex (PIC) was not biochemically different from wild type virions and that the viral cDNA is processed for integration [10]. A similar mutant, p12 S61A, can also integrate *in vitro *[10], leading to the hypothesis that p12 may be important for nuclear import, nuclear retention or localization of the PIC to preferred integration sites in the host genome. LEDGF provides a chromosome tethering function for HIV-1 [11,12], but currently no protein has been identified with such a role for MLV.

Phosphorylation of p12 occurs during the latter stages of infection (mainly on S61) but this modification is not essential for early events, as viral revertants arise from the p12 SS(61,65)AA double mutant without recovering p12 phosphorylation [13,14]. However, Yueh and Goff highlighted the importance of four arginine residues in the C-terminus of p12, and suggested that the presence of positively charged residues was required for the early stages of replication [13]. Blocking the cleavage between p12 and CA prevents the formation of a β-hairpin at the N-terminus of CA and inhibits formation of the mature viral core [15]. Whilst this is lethal to the virus, blocking the separation of p12 from MA has only a minor effect on infectivity [16]. Nuclear magnetic resonance studies showed that p12 was unstructured in a fragment including the N-terminal domain of CA [17]. In this study, no long-range interactions between p12 and CA were observed, although such interactions have been reported for the p10 and CA proteins of Rous sarcoma virus in immature particles [18]. However, a cooperative effect between p12 and CA has been shown genetically by constructing chimeras between MLV and spleen necrosis virus (SNV) [19]. Infectious virus was only formed when the p12 protein (p18 in the case of SNV) was from the same virus as the CA protein. Furthermore, immunofluorescence analysis of infected cells showed that p12 co-localized with viral DNA and CA, implying that p12 is a functional component of the MLV PIC [20]. This study also described the movement of p12 during infection. During interphase, p12 is present primarily in the cytoplasm but during mitosis p12 is found in the proximity of the chromosomes. Most strikingly, p12 from a C-terminal mutant (PM14) did not display this accumulation at chromosomes during mitosis [20], suggesting that p12 may be involved in PIC localization.

It has recently been reported that MLV particles incorporate clathrin via a DLL motif within p12 [21]. The incorporation of clathrin into HIV-1 virions through interaction with integrase (IN) has also been reported, although the significance of this interaction is unknown [21,22]. However, knock down of clathrin in virus producer cells resulted in ~2 fold reduction in MLV infectivity, whereas mutation of the DLL motif in p12 reduced infectivity to <1% of wild type particles. This discrepancy may be due to an incomplete knock down of clathrin but it suggests that the DLL motif is important for another reason. The authors concluded that the loss of infectivity was due to a morphological defect in the MLV PIC that included loss of mature p12 [21].

Here, we demonstrate that p12 contains two domains that act in concert and can behave in a dominant negative manner. We show that these domains are conserved in a range of gammaretroviruses, but that the N-terminal domain is more variable and is less sensitive to single amino acid changes than the C-terminal domain. Nevertheless, the C-terminal domains of MLV and GaLV are interchangeable. We also show that purified p12 is monomeric in solution at high concentrations, eliminating oligomerisation as a mechanism for the dominant negative effects. Importantly, we identify potential functions for each domain and, based on these data, propose a model for p12 function during the early stages of retroviral replication.

## Results

### Mo-MLV vectors carrying mutations in p12 are non-infectious but mature p12 is incorporated into particles

**Figure 1:**
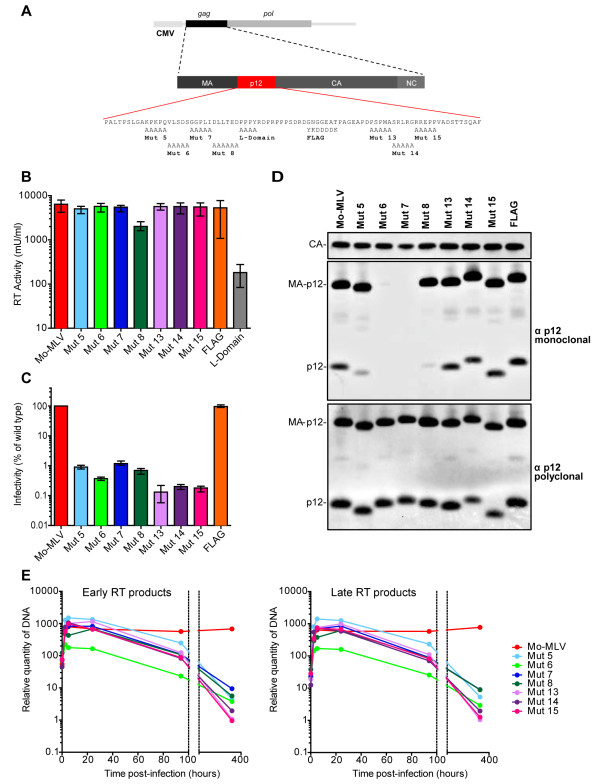
**Characterisation of p12 mutants in a Mo-MLV vector system.** (**A**) Schematic representation of the Mo-MLV Gag-Pol expression plasmid used in this study, showing the amino acid sequence of p12. The blocks of residues changed in each substitution mutant and the mutant names are indicated. (**B**) LacZ-encoding wild type or mutant Mo-MLV virus-like particles (VLPs) were produced in 293T cells by transient transfection and reverse transcriptase (RT) activity was quantified by a modified ELISA as a measure of virus production. Results show the mean and range of three independent experiments. (**C**) Equivalent RT units of VLPs from (B) were used to challenge D17 cells. Infectivity was measured by detection of β–galactosidase activity in a chemiluminescent reporter assay. Results are plotted as the percentage of infectivity compared to wild type Mo-MLV and show the mean and range of three independent experiments. (**D**) Immunoblot analysis of VLPs from (B) showing p12 (bottom panel) and capsid (top panel) protein processing. The middle panel shows that the CRL-1890 monoclonal antibody fails to recognize some mutant p12 proteins. (**E**) D17 cells were infected with wild type or mutant VLPs and total DNA was isolated at various times post infection as indicated. The relative amounts of minus strand strong stop (left panel) or second strand extension (right panel) were measured by qPCR. Results are representative of at least three independent experiments.

The p12 protein was shown to be important for early events in replication by studies introducing mutations into the Mo-MLV provirus [8]. In order to test the effects of these mutations in single cycle infections in a range of different cell lines, we cloned the same mutations as Yuan *et al*. into a Mo-MLV Gag-Pol vector (Figure 1A). VSV-G pseudotyped virus-like particles encoding LacZ were synthesized by co-transfection of 293T cells and viral titres were estimated using a modified ELISA for reverse transcriptase (RT) activity. Particle release was similar for all mutants except mutant 8 (Figure 1B). The release of mutant 8 particles was reduced approximately three fold. In this mutant, the residues immediately adjacent to the L-domain required for budding have been altered, and this may account for the reduction in titre. In comparison, mutating the L-domain itself reduced particle release 50–100 fold (Figure 1B, grey bar). When equivalent RT-units of these mutant VLPs were used to challenge canine D17 cells in a single cycle infectivity assay, the infectivity of every mutant was reduced 100–1000 fold compared to wild type VLPs (Figure 1C). In addition, the infectivity of a subset of the mutant VLPs was significantly reduced in a panel of different cell lines (Additional file 1). Replacing residues 46–52 with a FLAG epitope had no effect on particle production or infectivity (Figures 1B and 1C). These data are comparable with those seen for proviral mutants [8] and show that the block to infection is independent of the viral envelope and the route of entry and that the function of p12 is not species specific. 

To address whether these mutations affected processing of the Gag polyprotein, we performed immunoblotting experiments with lysed viral particles. We showed that both CA and p12 proteins were cleaved from the Gag polyprotein in all cases (Figure 1D, top and bottom panels respectively), implying that normal processing occurred. Interestingly, our monoclonal antibody against p12 failed to detect mutants 6 and 7 and only weakly detected mutants 5 and 8, preferentially detecting the mutant MA-p12 processing intermediate (Figure 1D, middle panel). This antibody was recently used to show that mature p12 was missing from viral particles containing mutations in the DLL motif, which is mutated in mutant 8 [21]. However, using a combination of monoclonal and polyclonal antibodies, we have shown that wild type levels of mature p12 are, in fact, incorporated into viral particles, although even wild type p12 is incompletely processed (Figure 1D, middle and bottom panels and [8,15,21]). Furthermore, our immunoblot analysis suggests that the p12 monoclonal antibody epitope is found between residues 15–30, and so poorly detects the p12 from mutant 8. Thus, none of the mutations in p12 affect p12 processing in our experiments and equal levels of mature p12 were found in all VLPs.

### Mo-MLV vectors carrying mutations in p12 fail to integrate their DNA

To determine the timing of the block to replication, we performed quantitative PCR experiments to detect both early (minus strand strong stop) and late (second strand extension) RT products (Figure 1E, left and right panels respectively). D17 cells were incubated with equal RT-units of DNase-treated VLPs and grown for two weeks. Aliquots of cells were removed at various times post infection, total cellular DNA was purified and the relative quantity of MLV DNA was measured. For wild type particles, the amount of both strong stop and second strand products increased with time to a maximum at around 6 hours post infection and then remained at this level for the course of the experiment (Figure 1E, red lines), implying that the viral DNA had stably integrated into the cellular genome. However, for all mutants, levels of viral DNA initially increased, but then declined to background levels by two weeks indicating that the block to replication was after reverse transcription but before integration. Only mutant 6 (light green line) showed reduced accumulation of linear viral DNA compared to wild type particles at all time points, demonstrating that reverse transcription was impaired by this mutation. Interestingly, in contrast to previous experiments in NIH-3T3 cells [8], mutant 8 viral particles were fully competent to synthesize linear viral DNA in D17 cells (dark green line), although this DNA failed to integrate. We observed the same phenotype in 293T cells (data not shown). This implies that mutant 8 particles are competent to reverse transcribe in at least some cell types and thus the timing of the block to infection for this mutant is probably concomitant with reverse transcription.

### Mixed mutant/wild type particles are infectious and reveal two functional domains

**Figure 2:**
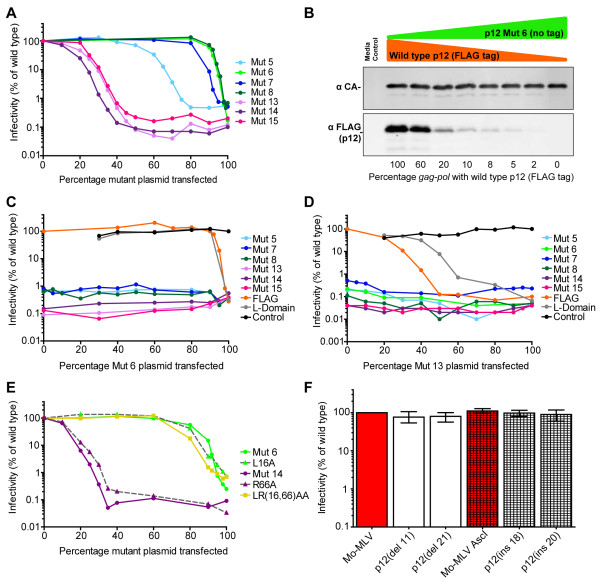
**Infectivity of mixed mutant/wild type p12 particles.** (**A**) Mo-MLV LacZ-encoding VLPs containing a mixture of wild type and mutant p12 were synthesized as described in the text. D17 cells were challenged with equal RT-units of VLPs and infectivity was determined by measuring β–galactosidase activity. Infectivity is plotted as a percentage of the infectivity of VLPs with 100% wild type p12. (**B**) Mixed particles containing various ratios of un-tagged mutant 6 p12 and FLAG-tagged wild type p12 were analyzed by immunoblotting with antibodies against MLV CA (top panel) and FLAG (bottom panel). (**C** and **D**) The infectivity of mixed particles containing two different p12 mutants was tested as in (**A**). Either mutant 6 (**C**) or mutant 13 (**D**) were mixed with each other mutant (shown in the key), wild type FLAG-tagged p12 (orange line) or an L-domain mutant (grey line). As a control for mixed particle synthesis, wild type p12 was mixed with an L-domain mutant (black line). For this sample, the percentage of wild type p12 plasmid in the transfection is plotted on the x-axis. (**E**) Mixed virus particles containing wild type p12 and either mutant 6, mutant 14, L16A, R66A or the double mutant LR(16, 66)AA were produced and analyzed as in (A). All panels are representative of 3 experiments. (**F**) Deletions of 11 or 21 amino acids (residues D43-P53 and P37-A57 respectively) were created in p12. Additionally, an *AscI* site was introduced into *gag* corresponding to residues 49 and 50 in p12 and an alanine cassette (of 18 or 20 amino acids) was inserted. VLPs bearing these alterations were produced and their infectivity tested in D17 cells. The mean and range of three independent experiments are shown.

As p12 comprises part of the Gag polyprotein, it is incorporated into viral particles with the same frequency as MA, CA and NC proteins. To investigate how much p12 is actually required for function, we assessed the infectivity of “mixed” viral particles that contained both mutant and wild type p12 proteins at varying ratios (Figure 2A). By using the Mo-MLV p12-FLAG construct to provide wild type p12, we confirmed that the amount of functional p12 in total viral particles decreased as the percentage of Mo-MLV p12-FLAG plasmid in the transfection mix decreased (Figure 2B). We also demonstrated that mixed particles were being produced by rescuing viral infectivity of the p12 substitution mutants by co-transfecting a Gag-Pol vector carrying an L-domain mutation that precludes viral particle production (Figure 2C and 2D, grey lines. Note that the absence of points when there is a high ratio of L-domain mutant in the transfection mix reflects the absence of virus production). Interestingly, analysis of the infectivity of mixed particles revealed that the phenotypes of p12 mutants fell into two groups with the exception of mutant 5 (Figure 2A). Excluding mutant 5, particles that contained p12 proteins with alterations to their N-termini (mutants 6, 7 and 8) remained infectious as the ratio of mutant to wild type p12 increased, until less than ten percent of the p12 in the particle was wild type. This implies that only a small amount of p12 is actually required for function. Conversely, particles that contained p12 proteins with alterations to their C-termini (mutants 13, 14 and 15) lost infectivity rapidly as the ratio of mutant to wild type p12 increased, so that particles had less than 0.5% infectivity when 50 percent of the p12 in the transfection was wild type. This is indicative of a dominant negative mutation. Particles synthesized with the construct carrying mutant 5 had a third phenotype: Infectivity correlated with the amount of wild type p12 present in the particle until 50% of the p12 was wild type, whereafter infectivity rapidly declined so that when 20% of the p12 was wild type the particles were no longer infectious.

As modifications N- and C-terminal of the L-domain had such distinct phenotypes, and previous studies had shown that a large region in the middle of p12 was not sensitive to mutation [8,9], this suggested that there were two functional domains in p12. To investigate whether the two potential domains functioned independently of one another, we made mixed particles with pairs of mutant p12 constructs. We chose mutant 6 as an N-terminal mutant (Figure 2C) and mutant 13 as a C-terminal mutant (Figure 2D), and combined them with every other mutant in turn. Although we could rescue infectivity with either FLAG-tagged wild type p12 (orange line) or p12 with an L-domain mutation (grey line), none of the other p12 mutants could rescue the infectivity of either mutant 6 or mutant 13, suggesting that the two halves of p12 do not function independently. To investigate which of the two main phenotypes was dominant we engineered a double mutant that contained point mutations at both the N-terminus (L16A) and the C-terminus (R66A). Each point mutation individually reduces particle infectivity by at least 99% (Figure 2E) and shows the expected phenotype for an N- or C-terminal mutant in a mixed particle assay (Figure 2E). In the same experiment, the double mutant clearly exhibited an “N-terminal phenotype”, requiring only a fraction of the p12 to be wild type for full infectivity and displaying no dominant negative activity (Figure 2E, yellow line), confirming that the N-terminus must be functional in order to produce the dominant negative phenotype of the C-terminal mutants.

To determine whether the spacing between the domains was important, we deleted or inserted residues in our Gag-Pol plasmid at positions corresponding to the “inter-domain region” of p12 and tested the infectivity of particles made from these vectors (Figure 2F). Deleting 11 or 21 amino acids, from residues D43-P53 and P37-A57 respectively, or inserting either 18 or 20 extra amino acids between residues 49 and 50 had no effect on viral infectivity (Figure 2F), implying the two domains do not have to be a set distance apart to function.

### Similar mutations in other gammaretroviruses inhibit infectivity

**Figure 3:**
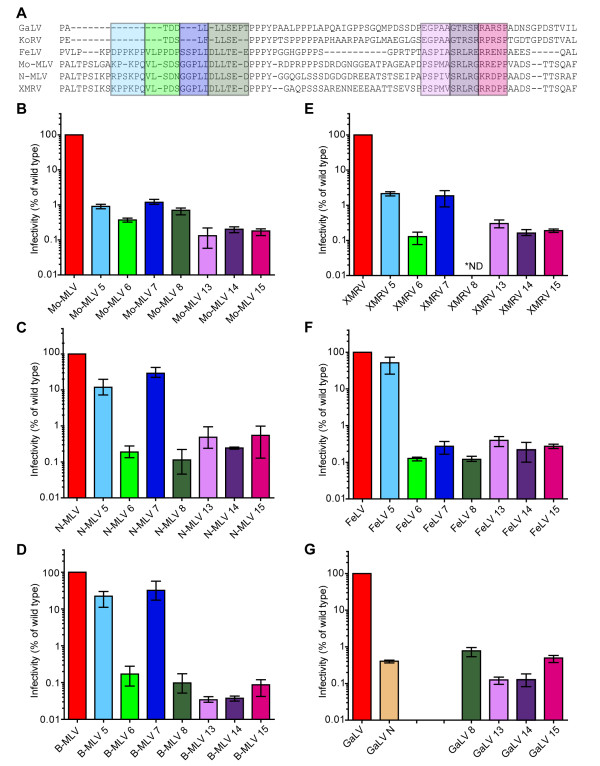
**The effects of p12 mutations in different gammaretroviruses.** (**A**) An alignment of the p12 sequence from various gammaretroviruses is shown. Shaded boxes indicate the position of each p12 mutant in each species and colours correspond to the bar colours in the graphs below. As GaLV has a small N- terminal region, the mutant TDDLL/AAAAA has been named GaLV N (**G**). (**B**-**G**) LacZ-encoding wild type or mutant VLPs were produced for each species of gammaretrovirus and equivalent RT-units of VLPs were used to challenge D17 cells. Infectivity was measured by detection of β–galactosidase activity in a chemiluminescent reporter assay. Results are plotted as the percentage of infectivity compared to the infectivity of the corresponding wild type VLPs for each species. The mean and range of three independent experiments are shown.

In order to examine the function of p12 in other gammaretroviruses, we first aligned the Gag sequences of N-tropic MLV (N-MLV), xenotropic murine leukaemia virus-related virus (XMRV), feline leukaemia virus (FeLV), koala retrovirus (KoRV) and gibbon ape leukaemia virus (GaLV) with Mo-MLV (Figure 3A). Aligning the p12 sequences at the PPPY motif revealed that KoRV and GaLV had very short N-terminal regions and FeLV had an 11 amino acid deletion in the middle of the protein. The most closely related sequences were N-MLV and XMRV with 73% identity. Unsurprisingly, the highest sequence diversity between viruses was seen immediately after the PPPY motif where mutations in Mo-MLV did not affect infectivity (Figure 1C, orange bar, 2F and [8,9,20]). 

Based on these alignments, we made similar alanine substitution mutations to Mo-MLV mutants 5–8 and 13–15 (Figure 3A, coloured boxes) in N-MLV, B-tropic MLV (B-MLV), XMRV, FeLV and GaLV Gag-Pol plasmids, synthesized LacZ expressing, VSV-G pseudotyped VLPs and tested the infectivity of these particles in D17 cells (Figure 3B-G). Of note, the sequence of B-MLV p12 is identical to N-MLV p12 (Figure 3A); and despite our best efforts, we were unable to clone XMRV mutant 8 (Figure 3E). As the N-terminus of GaLV was short, we mutated TDDLL to AAAAA and named this “mutant N” rather than mutant 5, 6 or 7 (Figure 3G). The results of altering the C-terminal region of p12 were more readily interpretable than those of the N-terminal mutants. For all five viruses, mutants 13, 14 and 15 were more than 100 fold less infectious than wild type viruses, highlighting the importance of this region of p12. However, the infectivity of N-terminal mutants varied with different viruses. In all cases, the infectivity of mutants 6 and 8 was less than 1% that of wild type virions as was the infectivity of GaLV mutant N. In contrast, the infectivity of mutant 5 was only reduced to 12%, 22% and 51% that of wild type for N-MLV, B-MLV and FeLV respectively, and the infectivity of mutant 7 was only reduced to 29% and 32% that of wild type for N-MLV and B-MLV respectively. The infectivity of FeLV mutant 7 was less than 1% that of wild type FeLV and the infectivities of XMRV mutants 5 and 7, although not reduced as much as the other XMRV mutants, were less than 2% that of wild type XMRV infectivity. The differences in the effects of these mutations were surprising because the sequence altered in mutant 5 is highly conserved between Mo-, N- and B-MLV, XMRV and FeLV, and the region modified in mutant 7 is identical between Mo-, N- and B-MLV and XMRV (Figure 3A). This suggests that the importance of individual amino acids depends on the context. Nevertheless, the requirement for the N- and C-terminal domains of p12 during infection is conserved for gammaretroviruses, although, in contrast to the C-terminal domain, the sequence of the N-terminal domain appears to be less sensitive to mutation.

### N-terminal p12 mutants cannot saturate TRIM5alpha or Fv1 restriction

**Figure 4:**
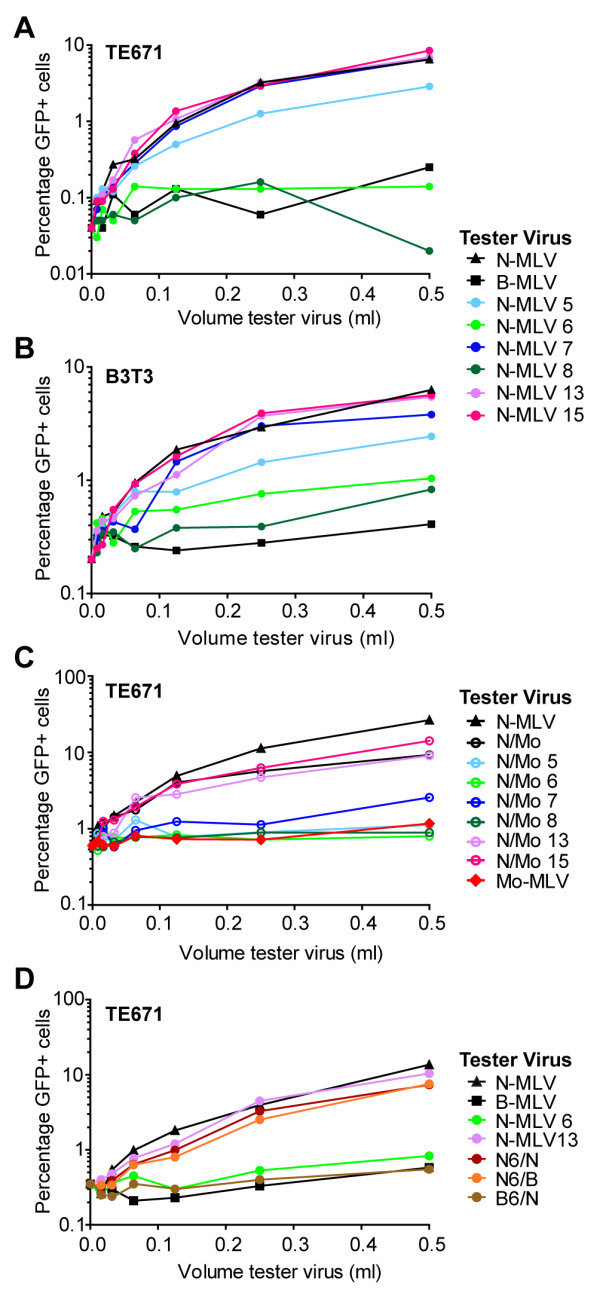
**The ability of p12 mutants to saturate TRIM5alpha and Fv1 activity.** (**A** and **B**) Supernatants from 293T cells containing LacZ-encoding N-tropic MLV (N-MLV) tester viruses, with or without p12 mutations, or B-MLV were serially diluted 2-fold and used to challenge TE671 cells, which express TRIM5alpha (**A**), or B3T3 cells, that express Fv1^b^ (**B**). After 4 to 6 hours, the cells were infected a second time with a fixed dose of GFP-encoding N-MLV reporter virus. The percentage of GFP positive cells is plotted for each volume of tester virus. (**C**) Mo-MLV p12 mutants were converted to N-tropic viruses by changing 3 residues in CA. These mutants (named N/Mo) were used as tester viruses in TE671 cells as described in (**A**). (**D**) Mixed particles that contained a mixture of N- or B-tropic CA in addition to mutated and wild type p12 were synthesized as follows: 90% N-MLV mutant 6 with 10% wild type B-MLV (N6/B, orange line), 90% B-MLV mutant 6 with 10% wild type N-MLV (B6/N, brown line) and 90% N-MLV mutant 6 with 10% wild type N-MLV (N6/N, dark red line). These viruses were used to test abrogation of TRIM5alpha restriction in TE671 cells as described in (**A**). All graphs are representative of at least three independent experiments.

Mutation of p12 results in inhibition of viral replication at stages of the life cycle reminiscent of the blocks imposed by the cellular restriction factors TRIM5alpha and Fv1 (prior to reverse transcription and integration respectively) [2]. The viral target for these restriction factors is the mature CA protein [2] and it has been proposed that p12 is functionally connected to CA [19]. We, therefore, wondered whether mutating p12 would impact the ability of VLPs to saturate these restriction factors, and by synthesizing N- and B-MLV p12 mutants (Figure 3) we were able to test this. We added increasing amounts of LacZ-encoding N-MLV tester virus carrying p12 mutations or wild type N- or B-MLV controls, followed by a fixed and equal amount of GFP-encoding wild type N-MLV reporter virus to either TE671 cells (Figure 4A) or B3T3 cells (Figure 4B) that express TRIM5alpha or Fv1^b^ respectively. The percentage of cells expressing GFP was measured three days post infection. If the tester virus can be recognized by the restriction factor, then adding increasing amounts of virus will saturate the factor, allowing the second infection by the GFP reporter virus to proceed without restriction. Thus, prior exposure of the cells to the restricted N-MLV tester virus enabled the N-MLV GFP reporter virus to efficiently infect both TE671 and B3T3 cells (Figure 4A and 4B, black triangles) whereas prior exposure to B-MLV, which is not restricted in these cells, had no effect on the reporter virus infection (black squares). Figures 4A and 4B show that in both cell types, mutants 13 and 15 were able to abrogate restriction as well as wild type N-MLV. This indicates that although these mutants are non-infectious, the restriction factors are able to recognize them, in turn implying that their CA shells are fully processed, assembled and present in an accessible compartment of the cell. Mutant 7 was also able to saturate both restriction factors as well as wild type N-MLV, although this was less surprising because this mutation in N-MLV only moderately reduces infectivity (Figure 3C and Additional file 2A). Mutant 5 was able to partly saturate both factors (Figure 4A and 4B), which may reflect the level of infectivity of this mutant (Figure 3C and Additional file 2A). N-MLV mutants 6 and 8 were unable to abrogate TRIM5alpha restriction and only had a minor effect on Fv1^b^ restriction, suggesting that the restriction factors cannot interact with these particles (Figure 4A and 4B). Therefore, the different mutations in p12 result in different abilities to saturate retroviral restriction factors. The same trends were seen in NIH-3T3 cells with B-MLV p12 mutants (data not shown). 

As N-MLV mutants 5 and 7 were relatively infectious, we could not extrapolate the results to the defective Mo-MLV mutants 5 and 7 and draw a general conclusion about the abrogation ability of the N-terminal mutants. It was possible that the failure to abrogate restriction was linked to the defect in reverse transcription rather than a general N-terminus property. Unfortunately, a high background prevented us from measuring the levels of cDNA produced from either Mo-MLV or N-MLV in TE671 or NIH-3T3 cells, but we were able to measure the ability of N-MLV to reverse transcribe in D17 cells (Additional file 3). All of the N-MLV mutants appeared to reverse transcribe in these cells, including N-MLV mutant 6, consistent with the notion that the precise timing of the block varies with virus and cell type and suggesting that the failure to saturate restriction factors was not due to a failure to reverse transcribe. However, to address this fully, we made mutations in the CA region of the *gag* gene for each of our Mo-MLV mutants that result in three amino acid changes in CA; D82N, A110R and H117L, and converts Mo-MLV into an N-tropic virus (labelled N/Mo) [23]. The conversion of these residues did not alter the infectivity of any of the VLPs in D17 cells (Additional file 2B). In saturation assays, the N/Mo VLPs with wild type p12 or any of the C-terminal changes were able to saturate TRIM5alpha in TE671 cells nearly as well as wild type N-MLV (Figure 4C). Conversely, the N/Mo VLPs carrying alterations to the N-terminus of p12 all failed to abrogate TRIM5alpha restriction (Figure 4C).

There are a number of potential reasons why the N-terminal mutants are unable to saturate TRIM5alpha. One is that these mutations in the p12 region of *gag* affect the production of functional CA protein and this prevents the particles from being recognized. In order to address this, we performed saturation assays in TE671 cells with mixed particles that contained a mixture of N- and B-tropic CA in addition to mutated and wild type p12 (Figure 4D). Particles were synthesized by mixing Gag-Pol expression plasmids as follows: 90% N-MLV mutant 6 with 10% wild type B-MLV (N6/B), 90% B-MLV mutant 6 with 10% wild type N-MLV (B6/N) or 90% N-MLV mutant 6 with 10% wild type N-MLV (N6/N). As seen in previous mixed particle experiments with N-terminal mutants (Figure 2A), including 10% wild type p12 in the transfection mixes ensured that all viruses were fully infectious in D17 cells (Additional file 2C). Of the three mixed particles, N6/B, and N6/N were able to saturate TRIM5alpha as well as wild type N-MLV and N-MLV mutant 13, despite 90% of the CA protein being produced from a *gag* gene carrying mutations in p12 (Figure 4D, orange and dark red lines). Moreover, all of the N-tropic CA in N6/B was produced from a mutated *gag* gene, implying that this CA protein is fully competent to interact with TRIM5alpha. In contrast, the B6/N particles were unable to saturate TRIM5alpha as they only contained 10% N-tropic CA and so would not be recognized by the restriction factor. We saw the same effect when we made similar mixed particles with N-MLV mutant 8 and when we infected B3T3 cells with either virus set (data not shown). Thus, it seems that mutations in the p12 region of *gag* do not cause aberrant CA function and the inability of the N-terminal mutants to saturate restriction factors is likely attributed to the loss of p12 function directly. This implies that the N-terminal p12 mutants are already defective before restriction factor binding, ie very early in infection, and suggests a role for p12 in core stability or localization.

### Mutations in the C-terminus of p12 can be rescued by a chromatin binding motif

**Figure 5:**
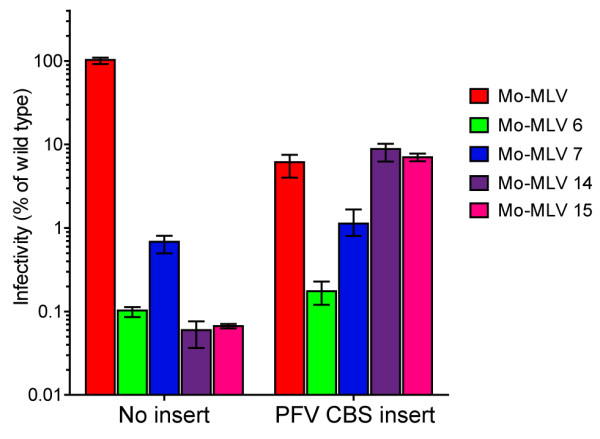
**The effect of inserting a chromatin binding domain into p12.** Sequence coding for the 14 amino acid PFV chromatin binding sequence (CBS) was inserted into the middle of p12 in Mo-MLV Gag-Pol expression vectors carrying wild type p12 or p12 mutants 6, 7, 14 or 15 following introduction of an *AscI* site. These plasmids were used to synthesize LacZ-encoding VLPs in 293T cells. D17 cells were challenged with equal RT-units of VLPs and infectivity was measured by detection of β–galactosidase activity in a chemiluminescent reporter assay. Infectivity is plotted as a percentage of the infectivity of Mo-MLV containing wild type p12 without the introduced *AscI* site. The mean and range of three independent experiments are shown.

Alterations to the C-terminus of p12 did not affect recognition of the viral core by restriction factors (Figure 4) and it is apparent that the functional requirement for the C-terminal domain is a later event in the viral life cycle than the requirement for the N-terminus (Figure 2E). Previous studies showed that during mitosis, the co-localization of p12 with chromatin was greatly reduced for a C-terminal mutant protein [20]. Moreover, although alterations to the C-terminus inhibited integration of viral cDNA *in vivo*, PICs derived from at least one mutant virus were competent to integrate *in vitro*[10], suggesting that p12 may function to locate the PIC to chromatin. We therefore introduced the 14 amino acid chromatin binding sequence (CBS) motif from prototype foamy virus (PFV) into the inter-domain region of Mo-MLV p12 mutants to attempt to rescue the block to infection [24,25]. Unexpectedly, inserting the CBS into wild type p12 reduced the infectivity of viral particles to 10% that of wild type virions (Figure 5, red bars), raising the possibility that the CBS changes the chromatin targeting of Mo-MLV and that it is suboptimal. Adding the CBS to p12 mutants 6 or 7 had no affect on the infectivity of the resulting VLPs (Figure 5). However, particles carrying either the C-terminal mutation 14 or 15 as well as the CBS were ~100 fold more infectious than mutant 14 or 15 virions alone (Figure 5, purple and pink bars), and were as infectious as wild type particles carrying the CBS. This striking result indicates that the CBS can rescue the defect seen in the C-terminal mutants, implying that the C-terminus of p12 has a chromatin binding function. 

### The C-terminus of p12 is more sensitive to single amino acid changes than the N-terminus

**Figure 6:**
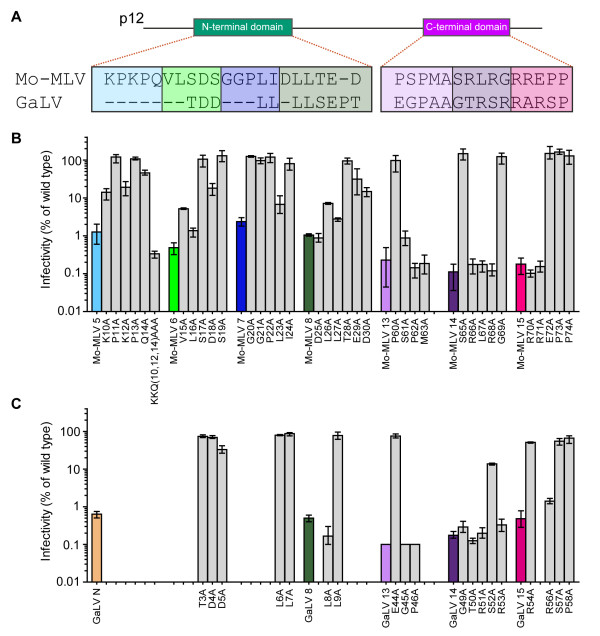
**Infectivity of Mo-MLV and GaLV with single amino acid changes in p12.** (**A**) An alignment of the residues comprising the N- and C-terminal domains of p12 in Mo-MLV and GaLV is shown. Colour shading indicates the original Mo-MLV p12 substitution mutants synthesized. (**B** and **C**) Single alanine substitutions were made for each residue within these domains for Mo-MLV p12 (**B**) or GaLV p12 (**C**). LacZ-encoding mutant VLPs were synthesized and equivalent RT-units of VLPs were used to challenge D17 cells. Infectivity was measured by detection of β–galactosidase activity in a chemiluminescent reporter assay. Results are plotted as the percentage of infectivity compared to the infectivity of the corresponding wild type VLPs for each species. The mean and range of three independent experiments are shown.

To more precisely map residues that comprise the functional domains at the N- and C-terminal regions of p12, each of the residues altered in the Mo-MLV alanine substitution mutants (Figure 6A) was changed to alanine and the infectivity of the resulting mutant particles was tested in a single cycle assay. Figure 6B shows the relative infectivity of the mutants to both wild type particles (set at 100%) and their “parental” alanine substitution mutant with stretches of 5–6 amino acids changed to alanine (coloured bars). Many individual changes had no effect on infectivity (changing residues P11, P13, S17, S19, G20, G21, P22, I24, T28, P60, S65, G69, E72, P73, P74) whilst, more surprisingly, some recapitulated the parental phenotype (L16, D25, S61, P62, M63, R66, L67, R68, R70, R71). The remaining mutations reduced infectivity by approximately 2-30 fold. The effect of altering single amino acids was most striking when changes were made at the C-terminal region of p12. Here, individual changes either had no effect on infectivity or reduced infectivity by >100 fold, to the levels seen with the parental mutants. One serine and the four arginine residues have previously been shown to be essential for infectivity [13], however the fact that the additional residues in between are also essential suggests that the run of amino acids from S61 to R71 may form an important motif. The effect of individual amino acid changes at the N-terminus of p12 was less pronounced. Several changes resulted in reduced viral infectivity compared to wild type virions, but not to the levels of the parental mutants, suggesting that the combination of residues is important. This is particularly obvious with mutant 5, where no individual change reduced infectivity more than 10 fold but a combination of three changes (K10, K12, Q14) reduced infectivity to <1% that of wild type virions (Figure 6B). Only six individual changes within the N-terminus of Mo-MLV reduced infectivity more than 10 fold: V15A, L16A, L23A, D25A, L26A and L27A. Strikingly, four out of the six residues were leucine. Two of these leucines (L26 and L27) together with D25 have recently been show to be a clathrin binding motif in p12 [21]. 

Comparing the sequences mutated in Mo-MLV mutants 5–8 and 13–15 with the other gammaretroviruses (Figure 3A, coloured boxes) revealed seven residues that were identical between all six viruses (in Mo-MLV numbering, D18, L23, L26, L27, E29, P62 and P74) together with several conservative changes. Of the identical residues, only P74 was shown to be completely dispensable for Mo-MLV p12 function in our alanine scanning experiment (Figure 6B). The D18A mutation in Mo-MLV reduced the viral infectivity to 20% that of wild type virions, however, the D25A mutation resulted in <1% wild type infectivity. Because the N-terminal regions of KoRV and GaLV are so short, it is possible that the asparagine residue(s) near the start of these sequences should really be aligned to D25 instead of D18 (Figure 3A). Therefore, to compare the residues in GaLV with Mo-MLV and assess the importance of each, we changed individual amino acids in GaLV p12 to alanine (Figure 6C). Interestingly, neither the D4A nor D5A changes affected infectivity more than three fold. Only one change in the N-terminus, L8A reduced infectivity >99%. This implies that the DLL motif thought to bind clathrin in Mo-MLV was not functional in GaLV, but also highlights the importance of leucines in the N-terminus of p12. Like Mo-MLV, several residues in the C-terminus of GaLV p12 appeared essential for function. Although not identical to Mo-MLV, a motif containing a proline and three arginines was also identified in GaLV p12 (Figure 6C). Taken together, it seems that the combination of amino acids is important in the N-terminus of p12 whereas individual amino acids constituting an arginine-rich motif are essential for the function of the C-terminus.

### The C-termini of Mo-MLV and GaLV p12 are interchangeable

**Figure 7:**
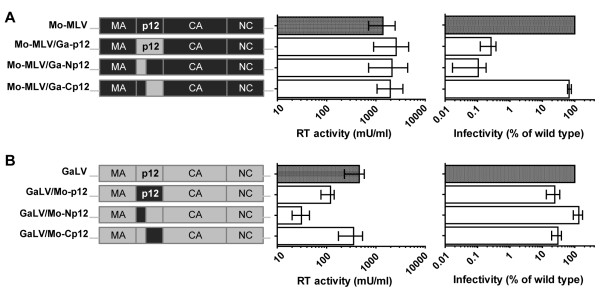
**Infectivity of Mo-MLV/GaLV p12 chimeras.** The diagrams on the left show the design of chimeric Gag-Pol plasmids based on (**A**) Mo-MLV with either the whole of p12, or just the N-terminus or C-terminus of p12 replaced with the p12 from GaLV, and (**B**) GaLV with either the whole of p12, or just the N-terminus or C-terminus of p12 replaced with the p12 from Mo-MLV. These chimeric Gag-Pol plasmids were used to produce LacZ-encoding mutant VLPs in 293T cells and RT activity was quantified by a modified ELISA as a measure of viral synthesis (middle panels). Equivalent RT-units of VLPs were used to challenge D17 cells and infectivity was measured by detection of β–galactosidase activity in a chemiluminescent reporter assay (right panels). Results are plotted as the percentage of infectivity compared to the infectivity of the corresponding wild type VLPs for each species. The mean and range of three independent experiments are shown.

To further compare the p12 proteins of Mo-MLV and GaLV, we engineered chimeras between the two. We replaced either the N-terminus, the C-terminus or the whole p12 sequence in Mo-MLV Gag with that from GaLV and vice versa (for nomenclature, see Figure 7, left hand panels), and synthesized LacZ-encoding VLPs. Although introducing all or part of GaLV p12 into Mo-MLV had no effect on particle production (Figure 7A, middle panel), introducing the N-terminus, and to a lesser extent full length, Mo-MLV p12 into GaLV reduced particle release as measured by RT activity in the producer cell supernatant (Figure 7B, middle panel). This is probably because the longer MLV p12 interferes with GaLV particle assembly. However, when particle titres were normalized, it was clear that Mo-MLV p12 could functionally replace GaLV p12 (Figure 7B, right panel), whereas only the C-terminus of GaLV p12 was interchangeable with Mo-MLV p12 (Figure 7A, right panel). This is not surprising, as the N-terminus of GaLV is very short compared to Mo-MLV, but it suggests that the N-terminus of p12 may interact with a viral factor as the combination of p12 and other viral proteins seems to be critical. To confirm that the chimeric Gag proteins were cleaved normally, we performed immunoblot analysis on viral particles (Additional file 4). Unfortunately, none of our anti-MLV antibodies recognized GaLV Gag proteins. However, by using a combination of our anti-MLV p12 monoclonal and polyclonal antibodies, that recognize the N- and C-terminus of Mo-MLV p12 respectively, we were able to detect cleaved p12 from all the chimeras that contained at least part of Mo-MLV p12 (Additional file 4). It did appear that the release of p12 from the MA-p12 precursor was slightly reduced for Mo-MLV/Ga-Np12 and GaLV/Mo-Np12, and, by inference from the reduction of free MA protein, in Mo-MLV/Ga-p12. However, the MA/p12 cleavage was also poor for wild type Mo-MLV (Additional file 4, Figure 1D and [8,15,21]). And as completely blocking the cleavage between MA and p12 only moderately reduces viral infectivity (less than 10 fold) [16], we think it is unlikely that this accounts for the inability of the N-terminus of GaLV p12 to functionally replace the N-terminus of Mo-MLV p12 (Figure 7A, right panel). 

### p12 does not self associate *in vitro*

**Figure 8:**
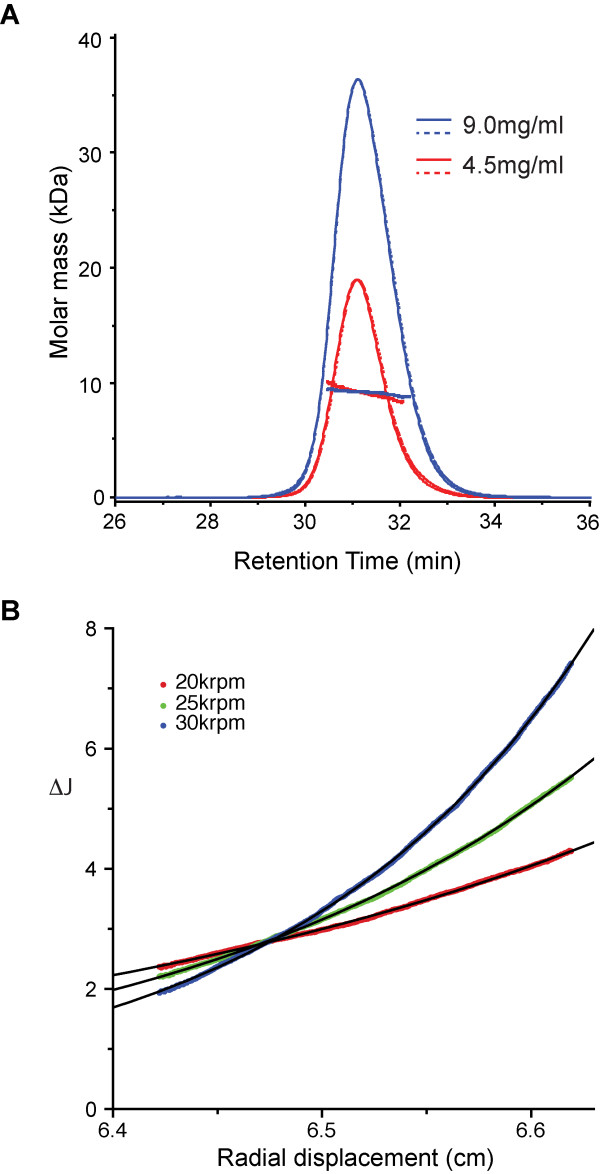
**Biophysical analysis of purified p12 protein. A**) SEC-MALLS analysis of p12. Scattered light intensity (solid line) and differential refractive index (dashed line) are plotted against retention time. Traces from p12 samples applied to a G75 (10/30) size exclusion column at 9.0 mg/ml (blue) and 4.5 mg/ml (red) are shown. The molar mass distributions, plotted as points, were determined throughout each peak as described in the text. **B**) Sedimentation equilibrium analysis of p12. A typical multispeed sedimentation equilibrium profile obtained from interference data collected on p12 at 100 μM is shown. The data were collected at 20, 25 and 30 krpm (red, green and blue points respectively). A global fit single-species ideal solution model incorporating data from all speeds and concentrations (black lines) gives a p12 molar mass of 9.3kD.

Mo-MLV p12 protein was purified from *E. coli* using a GST tag. The protein was soluble at pH 1.8 and when heated at 65°C, indicating either highly reversible renaturation/denaturation or that it has little tertiary structure at all, at least when unbound to interaction partners. Multi-angle laser light scattering (MALLS) and analytical ultracentrifugation (AUC) were used to determine that p12 was a single species (Figure 8A) with a narrow distribution of molar mass with no significant concentration dependency (Figure 8A). In order to confirm the MALLS data, quantitative sedimentation equilibrium experiments at multiple rotor speeds and varying sample concentration were undertaken (Figure 8B). No concentration dependency was apparent, so global fitting, incorporating the data from multiple speeds and multiple sample concentrations, was applied to extract a final weight-averaged molar mass of 9.3 ± 0.2kD (Figure 8B). The finding that p12 remains a monomer, even at high concentrations, is important information for constructing a model for p12 function, as these data demonstrate that the dominant negative phenotype of the C-terminal mutants is unlikely to result from p12 self-association.

## Discussion

The early post-entry stages of the retroviral life cycle are targets for several restriction factors and antiretroviral drugs [2,26]. However, many of these steps, such as uncoating, trafficking and nuclear localization are poorly defined. A better understanding of the processes and interactions that occur during early infection may lead to novel therapeutics for retroviral infections and improved retroviral vector design. In 1999, Yuan *et al*. showed that the p12 protein of MLV was essential during the early stages of MLV replication [8] and a later study identified “footprints” in p12 where insertions inhibited viral infectivity [9]. However, the function of p12 has remained obscure. Further studies showed no gross differences in PIC structure between wild type and mutant virions [10], and the mutant PICs tested were competent to integrate *in vitro*[10]. Although the protein appears to be phosphorylated in cells, this does not seem to be necessary for function [13].

To enable us to perform a variety of assays and infect multiple cell lines, we cloned the panel of p12 mutants synthesized by Yuan *et al*. into a Mo-MLV Gag-Pol vector and showed that the infectivity of the resulting VLPs was reduced to near background levels in a range of different cell lines (Figure 1C and Additional file 1). Particle production was only reduced for mutant 8 and Gag processing was normal for all mutants (Figure 1B and 1D). This implies that p12 function is independent of the viral envelope, route of entry and the viral gene-encoding RNA and it is not species specific, suggesting a more fundamental role during infection. Interestingly, particles with modifications to the N-terminus of p12 had different phenotypes to particles with alterations in the C-terminus of p12 (Figure 2A), suggesting that these regions formed separate “domains” with distinct functions. The length and composition of the linker between these domains could be modified without affecting viral infectivity (Figure 2F), but both domains had to be present on the same molecule to function (Figure 2C and 2D).

**Figure 9:**
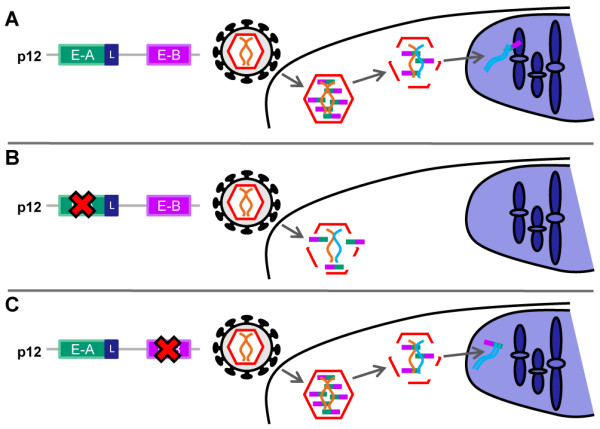
**Model for the function of p12 during the early stages of retroviral replication.** In addition to the late-domain, we propose that p12 carries two early domains in the N-terminus (Early-A, E-A, green box) and the C-terminus (Early-B, E-B, purple box). (**A**) Infection of a cell with a gammaretrovirus containing wild type p12 leads to successful integration of viral cDNA (turquoise) into the host chromatin. (**B**) Alterations to the N-terminal domain of p12, E-A, affect the stability of the viral core and abort infection very early in the replication pathway, sometimes inhibiting reverse transcription. The virus is therefore unable to abrogate restriction factors. (**C**) Alterations to the C-terminal domain of p12, E-B, do not affect the very early stages of infection and p12 is present in the PIC by virtue of interactions at the N-terminus. However, p12 is unable to tether the PIC to host chromatin and the viral cDNA cannot integrate successfully into the DNA of the host.

The dominant negative phenotype of the C-terminal mutants and the N-terminal-like phenotype of the double mutant in our mixed particle assays (Figure 2A and 2E), suggests that p12 makes important interactions at the N-terminus that are still able to occur when the C-terminus is defective, such that C-terminally modified p12 molecules compete with wild type p12 proteins. This implies that the N-terminus of p12 likely makes interactions before the C-terminus is required and/or binds a limiting factor. As we have shown that p12 does not self-associate *in vitro* at high concentrations (Figure 8), although with the caveat that the concentration of p12 in viral particles is possibly greater than in our experiments, we propose that p12 links two other factors together. It has been shown that p12 co-localizes with nascent viral cDNA and CA proteins in the viral PIC [20]. We hypothesize that p12 links the viral PIC to cellular chromatin. In this scenario, first the N-terminus of p12 would bind the PIC (the limiting factor) and then the C-terminus would bind chromatin once the nuclear envelope has broken down during mitosis, tethering the PIC to cellular DNA, ready for integration (Figure 9). 

By inserting the CBS from PFV [24,25] into p12 (Figure 5), we have shown that the defect imposed by changes to the C-terminus of p12 can be overcome by providing an alternative route for chromosome binding, although the CBS presumably doesn’t recapitulate the normal process of chromosome binding as adding this motif to wild type p12 reduces infectivity by approximately 90%. This could also explain why PICs from virions with defects in the C-terminus of p12 are competent to integrate into naked DNA *in vitro,* where locating and tethering target DNA is unlikely to be as complex [10]. Moreover, C-terminally defective PICs did not co-localize with mitotic chromatin in microscopic studies [20]. A chromatin-binding role for the C-terminus of p12 would also fit with the fact that its function seems to be conserved in other gammaretroviruses (Figure 3) and especially with our findings that the C-terminus of GaLV and MLV are functionally exchangeable (Figure 7). Our data, that even single amino acid changes in the C-terminus of p12 completely ablate function in both viruses (Figure 6), imply that the C-terminus harbors a binding motif. Although the amino acid sequence is not perfectly conserved across gammaretroviruses, the requirement for a cluster of arginines appears constant (Figure 3A). It is tempting to speculate that p12 may provide the function for gammaretroviruses that LEDGF provides for HIV [11,12], especially when it was reported over 30 years ago that p12 has sequence homology with histone H5 [27], albeit at the N-terminus, but further work is required to determine whether the interaction with chromatin is direct or occurs via an intermediate factor.

The function of the N-terminus of p12 appears to be more complex. Although changing 5–6 amino acids to alanine within a 21 amino acid stretch is detrimental to Mo-MLV, not all of these changes inhibit function in other gammaretroviruses (Figure 3). In particular, the residues altered in mutant 7 are identical between Mo-MLV and N-MLV and yet mutating them in Mo-MLV leads to ~99% reduction in infectivity (Figure 3B, dark blue bar) whilst the same mutation in N-MLV only reduces infectivity three fold (Figure 3C, dark blue bar). Therefore, the context of the amino acids seems to be important. We propose that the N-terminus of p12 binds to a viral component of the PIC, accounting for the different requirement for particular residues in p12 depending on what the specific binding target is. The specificity of the interaction may also explain why the N-terminus of Mo-MLV p12 can replace the function of the N-terminus of p12 in GaLV (Figure 7B), but the GaLV p12 cannot replace that of Mo-MLV (Figure 7A). Moreover, for most of the residues in the N-terminus of both Mo-MLV and GaLV, single alanine substitutions had little effect on viral infectivity (Figure 6). For example, the K10A, K12A and Q14A changes in Mo-MLV individually reduce infectivity <90%. However, making all three changes together reduces infectivity >99.5%. This leads us to hypothesize that the N-terminus of p12 makes multiple weak interactions that act in concert to form a strong interaction, perhaps binding a large surface. The CA shell of the viral core would be a possible example of such a surface and it has been proposed that the restriction factors Fv1 and TRIM5alpha bind this surface through multiple weak interactions [28].

Based on the results of the restriction factor saturation assays (Figure 4) it is clear that mutations in the N-terminus of p12 affect the ability of the restriction factors to recognize the CA shell. One way this could happen is if p12 binding normally stabilizes the CA shell. Alterations to p12 could prevent this stabilization, causing the core to breakdown prematurely before restriction factor binding. Alternatively, mutant p12 could still bind to the core but somehow prevent access by the restriction factor, although it is not clear how this would inhibit viral infectivity. Finally, mutations in p12 could alter the localization of the viral core in the cell, taking it to a location not accessible to restriction factors, nor conducive to a productive infection. Such compartmentalization has not been reported for retroviruses or restriction factors. Thus, our favored theory is that p12 helps to stabilize the viral core, perhaps even regulating the uncoating process. It has been suggested that uncoating is linked to reverse transcription [29] so, if the different mutants have different effects on the kinetics of uncoating, this may also affect the ability of the virus to reverse transcribe. This could explain how some N-terminal p12 variants affect reverse transcription in cells (Figure 1E), without affecting RT activity in an exogenous setting (Figure 1B). The precise timings of these events may be cell-specific, accounting for why the timing of the block varies for mutant 8 (Figure 1E, dark green line and [8]). However, TRIM5alpha is thought to bind to the core very early after membrane fusion before reverse transcription [30] so if particles carrying N-terminal p12 changes are already defective at this early stage, it is notable that reverse transcription takes place in most cases. Indeed, further studies on p12 may help elucidate the relationship between reverse transcription and uncoating.

It is not clear whether binding the CA shell would be sufficient to couple p12 to the PIC or whether the N-terminus of p12 would need to make additional interactions in order to perform its chromosomal tethering function. It has been shown that the DLL motif in the N-terminus interacts with clathrin [21], although the significance of this interaction remains unknown. The authors claimed that mutating this motif led to defects in p12 processing. However, we have shown that this may be an artefact of using particular antibodies for their study and clearly show that the p12 in mutant 8, in which the DLL motif is changed to AAA, is fully processed and incorporated normally into particles (Figure 1D). As the DLL motif is present in the other p12 mutants, this cannot account for the loss of function of most of the mutants.

Finally, the mixed particle experiments (Figure 2) suggest that only a fraction of the p12 in a viral particle is required to have a functional N-terminus, and therefore, by extrapolation from the fact that both domains must function on the same molecule (Figure 2C and 2D), only a small amount of active p12 is required for function. It seems reasonable that a limited amount of p12 would bind to a PIC (that contains only a subset of viral proteins and a single double stranded DNA) in order to tether it to chromatin, but further work is required to show how such a small amount of p12 could stabilize the CA shell. It is somewhat surprising that the cleavage of MA and p12 appears to be inefficient in wild type virions (Figure 1D and [8,15,21]). Indeed, this may be explained by the requirement for only a small amount of p12. Moreover, mutations that result in inhibiting this cleavage event only moderately affect viral infectivity [16], suggesting that p12 may retain much of its function even when fused to MA. In this scenario, a small amount of MA may be associated with the PIC.

## Conclusions

Prior to our study, the function of p12 during the early stages of MLV replication was unknown. Although there are undoubtedly other possible explanations for some of our results, we would like to propose a model for p12 function as shown in Figure 9: After Gag processing, the N-terminus of a proportion of the p12 protein in the particle interacts with the CA shell surrounding the viral core to regulate the uncoating process and allow reverse transcription to proceed. The p12 protein remains associated with the PIC as it traffics to the nucleus of the cell. As the cell enters mitosis and the nuclear envelope breaks down, the C-terminus of p12 interacts with cellular chromatin, targeting the PIC to the appropriate location for integration to occur and ensuring that the viral DNA is retained in the nucleus as the nuclear membrane reforms.

We hope that it will now be possible to test this hypothesis further and finally confirm the function(s) of the last remaining MLV Gag protein to be characterized.

## Methods

### Plasmids and cloning

To synthesize VLPs, three plasmids were co-transfected into 293T cells: An envelope expression construct for the vesicular stomatitis virus G protein (pczVSV-G) [31], a Mo-MLV-based retroviral vector encoding LacZ (pczLTR-LacZ) [32] or eGFP (pLNCG) [31,33] and a Gag-Pol expression plasmid. Wild type Mo-MLV Gag-Pol (named Mo-MLV) was expressed from pKB4, a vector synthesized by cloning the *gag-pol* region from pMD-MLV Gag-Pol [32] into pcDNA3.1, described previously [34]. N- and B-tropic MLV Gag-Pol expression vectors (pCI G3N and pCI G3B) have been described [31]. HG1 is a replication-incompetent XMRV clone in the pcDNA3.1 mammalian expression vector and has been described previously [34,35]. A stop codon was introduced into the env sequence at nucleotide 76–78 by site directed mutagenesis using primers: *for* 5’-gataattatggggatctaggtgagggcaggagcct and *rev* 5’- aggctcctgccctcacctagatccccataattatc to make the plasmid HG1-env. To produce a FeLV Gag-Pol expression plasmid, the FeLV *gag-pol* sequence from pczFeLVgp (a gift from J. Stoye) was cloned into pcDNA3.1 using the *Nhe*I and *Not*I restriction enzymes, and named pDJW1. GaLV Gag-Pol was expressed from a pcDNA3.1-based vector, pczGaLVgp (a gift from J. Stoye).

All Mo-MLV 5–6 amino acid alanine substitution mutants were generated by replacing the *BsrGI-XhoI* fragment from pKB4 with the corresponding mutated p12 fragments from proviral p12 mutants pNCS-PM5, -PM6, -PM7, -PM8, -PM13, -PM14, -PM15, -FLAG and –L domain (gifts from S. Goff [8]) creating a pKB series of mutants (pKB5, 6, 7, 8, 13, 14 and 15). Similar alanine substitution mutations were introduced into the p12 regions of N-tropic MLV, B-tropic MLV, XMRV, FeLV and GaLV by site directed mutagenesis of pCI G3N, pCI G3B, HG1-env, pDJW1 and pczGaLVgp respectively using the QuikChange kit (Stratagene). The primer sequences for each mutant are given in Additional file 5. Single amino acid changes were also introduced into Mo-MLV and GaLV by site directed mutagenesis of pKB4 and pczGaLVgp. The primer sequences for each mutant are given in Additional file 6. Additionally, deletions in p12 were generated by site directed mutagenesis of pKB4. Residues D43-P53 in p12 were removed using primers: *for* 5’-ccacccccttccgcgggagaggca and *rev* 5’-tgcctctcccgcggaagggggtgg, and residues P37-A57 were removed using primers: *for* 5’-gccttatagggacccggacccctccc and *rev* 5’-gggaggggtccgggtccctataaggc. To make insertions in p12, a unique *AscI* restriction site was first introduced into the p12 sequence in pKB4, pKB6, pKB7, pKB14 or pKB15 by site directed mutagenesis, generating pKB-AscI vectors. The mutations t144c, g146c, a147g, g148c and a149c were synthesized using primers: *for* 5’-cgacagggacggaaatggcgcgccagcgacccctgcgggag and *rev* 5'-ctcccgcaggggtcgctggcgcgccatttccgtccctgtcg. Linkers encoding the sequence to be inserted flanked by *AscI* sites were prepared by heating 4 μM complimentary oligonucleotides at 95°C for 5 minutes followed by annealing at 65°C for 10 minutes on a thermal cycler (Biorad). The annealed oligonucleotides were then cloned into the *AscI* site in the pKB-AscI vectors resulting in insertions between p12 residues 49 and 50 of: PA_15_GA (ins 18), PA_17_GA (ins 20) or the 14 amino acid CBS motif with a linker (P < NQGGYNLRPRTYQP > AG).

To convert wild type and p12 mutant Mo-MLV VLPs into N-tropic particles, mutations coding for three amino acid changes in CA; D82N, A110R and H117L, were introduced into the CA region of the appropriate wild type or mutant Mo-MLV Gag-Pol expression plasmid. These changes have previously been shown to make Mo-MLV particles sensitive to Fv1^b^[23]. The changes were made by site directed mutagenesis using the QuikChange kit (Stratagene) with the following primers: *D82N for* 5’-gcggtgcggggcaatgatgggcgcccc*, D82N rev* 5’-gggcgcccatcattgccccgcaccgc*, A110R for* 5’-gggattacaccacccagcgaggtaggaaccacctagt*, A110R rev* 5’-actaggtggttcctacctcgctgggtggtgtaatccc*, H117L for* 5’- ggtaggaaccacctagtcttgtatcgccagttgctcctagc*, H117L rev* 5’- gctaggagcaactggcgatacaagactaggtggttcctacc.

Chimeric p12 sequences were constructed by overlapping PCR from three DNA fragments amplified from either pKB4 (for Mo-MLV sequence) or pczGaLVgp (for GaLV sequence). All DNA fragments were amplified using Phusion DNA polymerase (Finnzymes) according to the manufacturer’s instructions. The amino acid sequence at the crossover junctions in the Mo-MLV based chimeras are as follows (i) *Mo-MLV/Ga-p12*: Mo-MLV MA(. SSLY/PALTDD.) GaLV p12 and GaLV p12(. DSTVIL/PLRA.) MoMLV CA; (ii) *Mo-MLV/Ga-Np12*: Mo-MLV MA(. SSLY/PALTDD.) GaLV p12 and GaLV p12(. LLSEPT/PPPY.) MoMLV p12; (iii) *Mo-MLV/Ga-Cp12*: Mo-MLV p12(. PPPY/PAALPP.) GaLV p12 and GaLV-p12(. DSTVIL/PLRA.)Mo-MLV CA. The final PCR fragments were amplified with primers *for* 5'-ccgcatggacacccagacca, and *rev* 5'-tggggcttctgcccgcgttt, and inserted into pKB4 between the *BsrGI* and *XhoI* sites. The amino acid sequences at the crossover junctions in the GaLV based chimeras are as follows: (i) *GaLV/Mo-p12:* GaLV MA(. PPIY/PALTPS.)Mo-MLV p12 and Mo-MLV p12-(. TTSQAF/PLRA.) GaLV CA; (ii) *GaLV/Mo-Np12:* GaLV MA(. PPIY/PALTPS.)Mo-MLV p12 and Mo-MLV p12(. EDPPPY/PAAL.) GaLV p12; (iii) *GaLV/Mo-Cp12:* GaLV p12(. SEPT/PPPYRD.) MoMLV p12 and Mo-MLV p12(. TTSQAF/PLRA.) GaLV CA. Due to the lack of suitable restriction sites in pczGaLVgp, a *BsrGI* site was introduced into this plasmid 5' of *gag*, and an *XhoI* site 3' of *pol* was removed, both by site directed mutagenesis. The final chimeric fragments were amplified using primers, *for* 5'-gcagatatccagcacagtgg, and *rev* 5'-cagaagacgctccctacctg, and inserted into pczGaLVgp between the introduced *BsrGI* site and a now unique *XhoI* site. The *BsrGI* site was then removed by site directed mutagenesis.

### Cells

All cell lines (293T, HeLa, TE671, Vero, MDBK, PK15, CF2TH, D17, MDCK, CRFK, CHO, CV-1, AH927, SIRC, B3T3, *M. dunni* and NIH-3T3) were maintained in DMEM (Invitrogen) supplemented with 10% heat inactivated foetal calf serum (Biosera) and 1% penicillin/streptomycin (Sigma), in a humidified incubator at 37°C and 5% CO_2_.

### VLP production

Virus-like particles of Mo-MLV, N-MLV, B-MLV, XMRV, FeLV and GaLV were prepared by co-transfection of 293T cells with a 1:1:1 ratio (or 2:1:1 for FeLV and 3:1:1 for GaLV) of three plasmids encoding the appropriate wild type or mutant Gag-Pol protein, VSV-G, and a reporter gene (β-galactosidase or GFP) respectively, using polyethylenimine (PEI, PolySciences) as a transfection reagent. To make mixed viral particles, wild type and mutant Gag-Pol expression plasmids (or two different mutants) were added to the transfection mix at different ratios, keeping the total concentration of Gag-Pol plasmid constant. After ~24 hours, cells were washed and fresh media was added for a further ~15 hours. Virus-containing supernatants were harvested, filtered, and viral titres were quantified using a modified ELISA for reverse transcriptase activity (Cavidi). In the restriction factor saturation assays, Turbofect (Fermentas) was used as transfection reagent. Approximately 18 hours after transfection, cells were washed and sodium butyrate media (0.02 M sodium butyrate, 10% FCS and 1% penicillin/streptomycin in DMEM) was added for 6 hours before replacing with fresh media. VLPs were harvested after 15 hours, as above.

### Infections

VLP infectivity was determined by challenging cells at ~40% confluency (D17 cells unless stated otherwise) with equivalent RT-units of VLPs. After 48–72 hours, cells were lysed and β-galactosidase activity in cell lysates was measured using the Galacto-Star system (Applied Biosystems).

For quantitative PCR analysis of viral cDNA, target cells were plated in 6-well plates, at a density of ~4x10^5^ cells per well, 1 day prior to infection. Equal RT-units of VLPs were treated with RQ1-DNase (Promega) for 1 hour at 37°C to remove any contaminating DNA from the virus preparation and then added to cells at 4°C. Cells were incubated for 2 hours on a shaker at 4°C, washed, resuspended in fresh DMEM and incubated at 37°C for a further 0–24 hours or 2 weeks. Total DNA was purified using the DNeasy Blood and Tissue kit (Qiagen) and analyzed by real-time quantitative PCR.

### Restriction factor saturation assays

TE671 cells expressing endogenous TRIM5alpha or B3T3 cells expressing endogenous Fv1^b^ were seeded at 10^5^ cells per well in a 12-well plate one day prior to infection. Cells were infected with 2 fold serial dilutions of freshly harvested 293T cell supernatants containing LacZ-encoding VLPs. Cultures were incubated for 4–6 hours at 37°C before adding a fixed amount of GFP-encoding N-MLV (equivalent to an MOI of 1 in *M. dunni* cells, resulting in ~30% GFP-positive *M. dunni* cells). After 72 hours, infected cells were harvested and the percentage of GFP-positive cells was determined by flow cytometry using a FACS Calibur analyzer (Becton Dickinson). For each experiment, LacZ-encoding B-tropic MLV was included as a negative control.

### Quantitative PCR analysis

Minus strand strong stop reverse transcription products were detected by amplification of the R-U3 region of Mo-MLV corresponding to nucleotides 997–1126 of the LacZ-LTR plasmid using primers oJWB45 (5’-GCGCCAGTCCTCCGATAGACTGA) and oJWB47 (5’-CTGACGGGTAGTCAATCACTCAG) with probe oJWB38 (5’-FAM-ATCCGACTCGTGGTCTCGCTGTTC-TAMRA) [36]. Second strand extension reverse transcription products were detected by amplification of the R-*gag* region corresponding to nucleotides 997–1240 of the LacZ-LTR plasmid using same forward primer, oJWB45, and probe oJWB38 but with the reverse primer oJWB48 (5’- ACAATCGGACAGACACAGATAAGTTG). Reactions were performed in triplicate, in TaqMan Gene Expression Master Mix (Applied Biosystems) using 900nM of each primer and 250nM of probe. Quantitative real time PCR was performed on a Fast 7500 PCR system (Applied Biosystems) using standard cycling conditions of 50°C for 2 minutes, 95°C for 10 minutes followed by 40 cycles of 95°C for 15 seconds and 60°C for 1 minute. The LacZ-LTR plasmid was diluted into purified DNA from the target cell line to create a series of samples that were used to calculate relative cDNA copy numbers and confirm the linearity of the assay.

### Immunoblot analysis

VLPs were concentrated by centrifugation through a 20% (w/v) sucrose cushion for 2–4 hours at 32,000 rpm at 4°C before immunoblot analysis. Viral CA and p12 proteins were detected using primary antibodies; rat anti-p30^CA^ monoclonal antibody (hybrodoma CRL-1912, ATCC), mouse anti-p12 monoclonal (hybrodoma CRL-1890, ATCC), goat anti-p12 polyclonal (a gift from J. Stoye), rabbit anti-FLAG polyclonal (sigma-aldrich) and secondary IRDye800CW conjugated antibodies (LI-COR Bioscience). Bands were visualized using the Li-cor Odyssey imaging and quantitation system (LI-COR Bioscience). MA was detected with rabbit anti-p15^MA^ primary antibody (a gift from A. Rein) and an anti-rabbit HRP conjugated secondary antibody using the Immobilon chemiluminescent substrate (Millipore) and hyperfilm processed with a Fijifilm FPM-3800A developer.

### GST-p12 protein purification

The p12 coding sequence was amplified from KB4 by PCR using primers: DA3f, 5’-AAGGATCCCCAGCCCTCACTCC, and KB131, 5’-CTACTCGAGTCAGAATGCCTGCGAGGTAGTG, and inserted into pGEX6.1 between the *BamHI* and *XhoI* sites to produce an N-terminally GST-tagged p12. The protein was expressed in *E. coli* BL21(DE3) by the addition of 1 mM isopropyl-β-D-thiogalactopyranoside (IPTG) to a mid-log culture. Cells were harvested and resuspended in 20 mM Tris pH7.7, 150 mM NaCl, 1 ml DTT (Buffer A) with addition of protease inhibitors and lysed by sonication. Clarified crude cell extracts were applied to a 5 ml GST-trap column (GE Healthcare). After washing with Buffer A, untagged p12 was eluted from the resin by digestion with precision protease over night. The eluate was then heated at 65°C for 5 minutes, centrifuged to remove precipitate and applied to a Superdex 75 (16/60) size exclusion column equilibrated in Buffer A. After size exclusion chromatography, fractions containing p12 were concentrated by ultrafiltration using Vivaspin centrifugal concentrators to ~10 mg/ml and stored frozen at −20°C. The purity of preparations was monitored by electrospray ionization mass spectroscopy and sodium dodecyl sulphate polyacrylamide gel electrophoresis (SDS-PAGE). Protein concentration was determined from the absorbance at 280 nm.

### SEC-MALLS

SEC-MALLS was used to determine the molar mass of p12. Typically, 100 μl protein samples were applied to a Superdex 75 10/300 GL column equilibrated in 20 mM Tris–HCl, 150 mM NaCl and 0.5 mM TCEP, pH 8.0, at a flow rate of 0.5 ml/min. The scattered light intensity and protein concentration of the column eluate were recorded using a DAWN-HELEOS laser photometer and an OPTILAB-rEX differential refractometer (dRI) (d*n*/d*c* = 0.186) respectively. The weight-averaged molecular mass of material contained in chromatographic peaks was determined from the combined data from both detectors using the ASTRA software version 5.1 (Wyatt Technology Corp., Santa Barbara, CA).

### Analytical ultracentrifugation

Sedimentation equilibrium experiments were performed in Beckman Optima XLI analytical ultracentrifuge using charcoal filled epon six-channel centrepieces in an An-50 Ti rotor. Prior to centrifugation, samples were dialyzed exhaustively against the buffer blank, 20 mM Tris–HCl, pH 7.5, 150 mM NaCl, 0.5 mM TCEP. After centrifugation for 18 h, interference data were collected at 2 hour intervals for 6 hours to ensure no further change in the profiles was observed. The rotor speed was then increased and the procedure repeated. Data were collected on samples of 50–200 μM of p12 at 20, 25 and 30krpm. The program SEDPHAT [37] was used to determine weight-averaged molar masses by non-linear fitting of individual multi-speed equilibrium profiles to a single-species ideal solution model.

## Competing interests

The authors declare that they have no competing interests.

## Authors’ contributions

All authors conducted the experiments and performed the analyses. DJW, VCB, IAT and KNB interpreted the results and wrote the manuscript. KNB conceived and designed the study and supervised the project. All authors read and approved the final manuscript.

## Supplementary Material

Additional file 1 **Activity of p12 mutants in a panel of cell lines.** Wild type Mo-MLV or p12 mutants 5, 6, 8 and 13 VLPs were produced in 293T cells by transient transfection and virus production was quantified by RT-ELISA. Equivalent RT-units of VLPs were used to challenge a panel of cell lines from different species and productive infection was measured after 48 hours by detection of β–galactosidase activity in a chemiluminescent reporter assay. Infectivity is reported as counts per second and the mean and range of three independent experiments are shown. Click here for file

Additional file 2 **Infectivity of particles used in TRIM5alpha and Fv1 saturation assays (Figure**4**).** (A) LacZ-encoding N-MLV tester viruses, with or without p12 mutations, or B-MLV were synthesized in 293T cells and used in TRIM5alpha and Fv1^b^ abrogation assays (Figure 4A and 4B). Equal RT-units of VLPs were used to challenge D17, TE671 and B3T3 cells and infectivity was measured by detection of β–galactosidase activity in a chemiluminescent reporter assay. Infectivity is plotted as a percentage of the B-MLV control. (B) The infectivity of N/Mo LacZ tester viruses that were used for TRIM5alpha abrogation assays (Figure 4C) was tested in D17 and TE671 cells as in (A). Infectivity is plotted as the percentage of the Mo-MLV control. (C) The infectivities of the mixed particles N6/B, B6/N and N6/N and the control viruses that were used for TRIM5alpha abrogation assays (Figure 4D) were tested in D17 and TE671 cells as in (A). Click here for file

Additional file 3 **Quantification of viral cDNA levels in D17 cells.** Wild type and mutant N-MLV VLPs were produced in 293T cells by transient transfection and equal RT-units of VLPs were used to challenge D17 cells. Total DNA was isolated at various times post infection as indicated, and the relative amounts of second strand extension were measured using qPCR. Results are representative of three independent experiments. Click here for file

Additional file 4 **Immunoblot analysis of Mo-MLV/GaLV chimeras.** LacZ-encoding Mo-MLV/GaLV p12 chimeric VLPs (A) and GaLV/Mo-MLV p12 chimeric VLPs (B) were produced by transfection of 293T cells (as in Figure 7). Equal RT-units of particles were concentrated through 20% (w/v) sucrose cushions and lysed in SDS loading dye. Viral proteins were separated on a 10% polyacrylamide gel by SDS-PAGE and p12 was detected with an anti-MLV p12 polyclonal antibody (A and B, top panels) and an anti-MLV p12 monoclonal antibody (A, middle panel; B, bottom panel). The approximate sizes of p12 and MA-p12 are indicated. GaLV p12 is not detected with either of these antibodies. In addition, the Mo-MLV based chimeras were probed with anti-MLV CA and anti-MLV MA antibodies (A, bottom panels). These antibodies did not cross react with GaLV Gag proteins. Click here for file

Additional file 5 Site directed mutagenesis primer sequences used to construct different gammaretroviral p12 alanine substitution mutations. Click here for file

Additional file 6 Site directed mutagenesis primer sequences used to construct single amino acid changes in p12. Click here for file
