# Supplementary figures and images for: The Gammaretroviral p12 protein has multiple domains that function during the early stages of replication
Source: Retrovirology. 2012 Oct 4;9:83. doi: 10.1186/1742-4690-9-83 (PMC3492146; doi:10.1186/1742-4690-9-83)

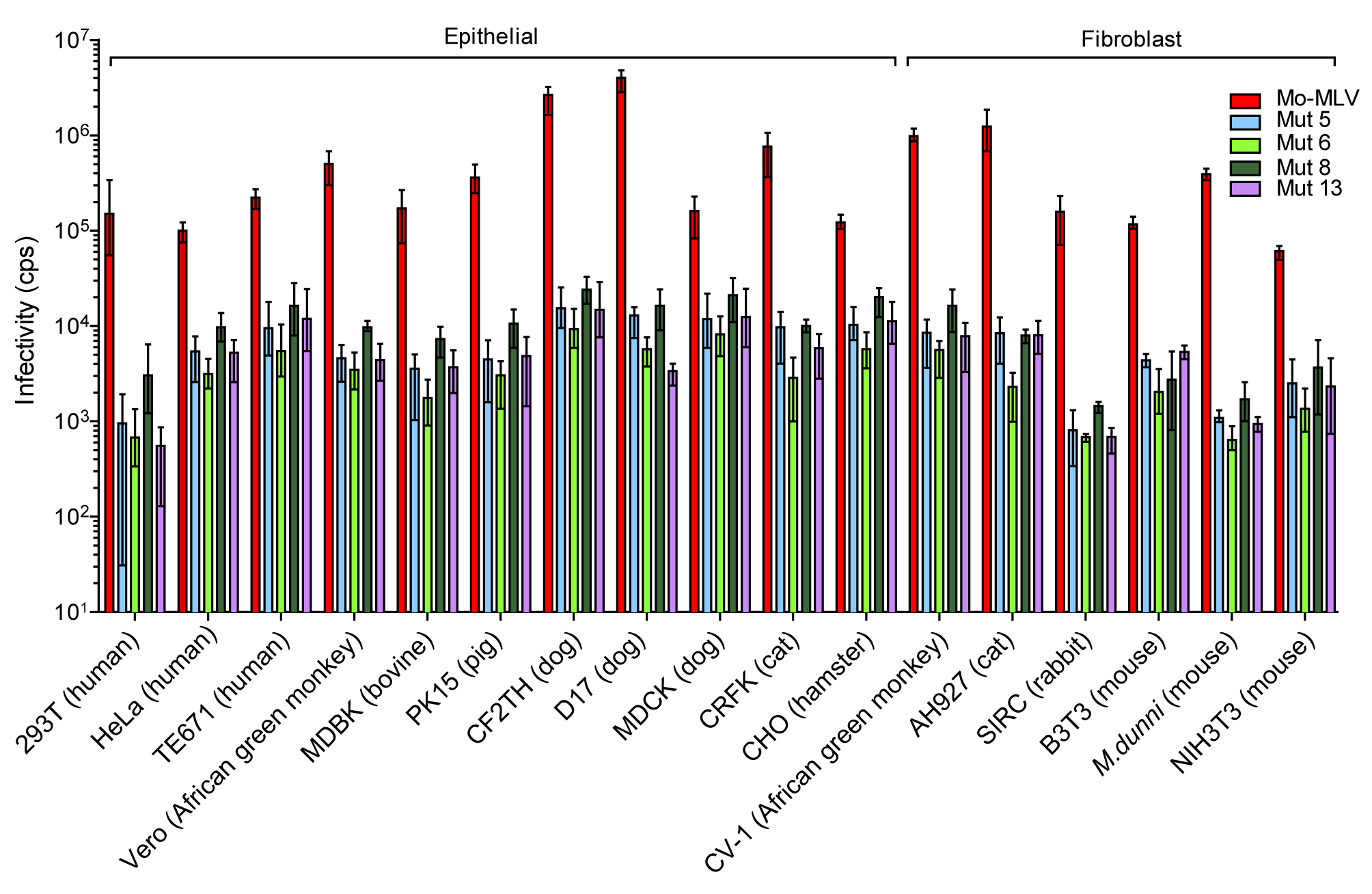

Supplement: Additional file 1 — Activity of p12 mutants in a panel of cell lines. Wild type Mo-MLV or p12 mutants 5, 6, 8 and 13 VLPs were produced in 293T cells by transient transfection and virus production was quantified by RT-ELISA. Equivalent RT-units of VLPs were used to challenge a panel of cell lines from different species and productive infection was measured after 48 hours by detection of β–galactosidase activity in a chemiluminescent reporter assay. Infectivity is reported as counts per second and the mean and range of three independent experiments are shown. [file 1742-4690-9-83-S1.tiff]

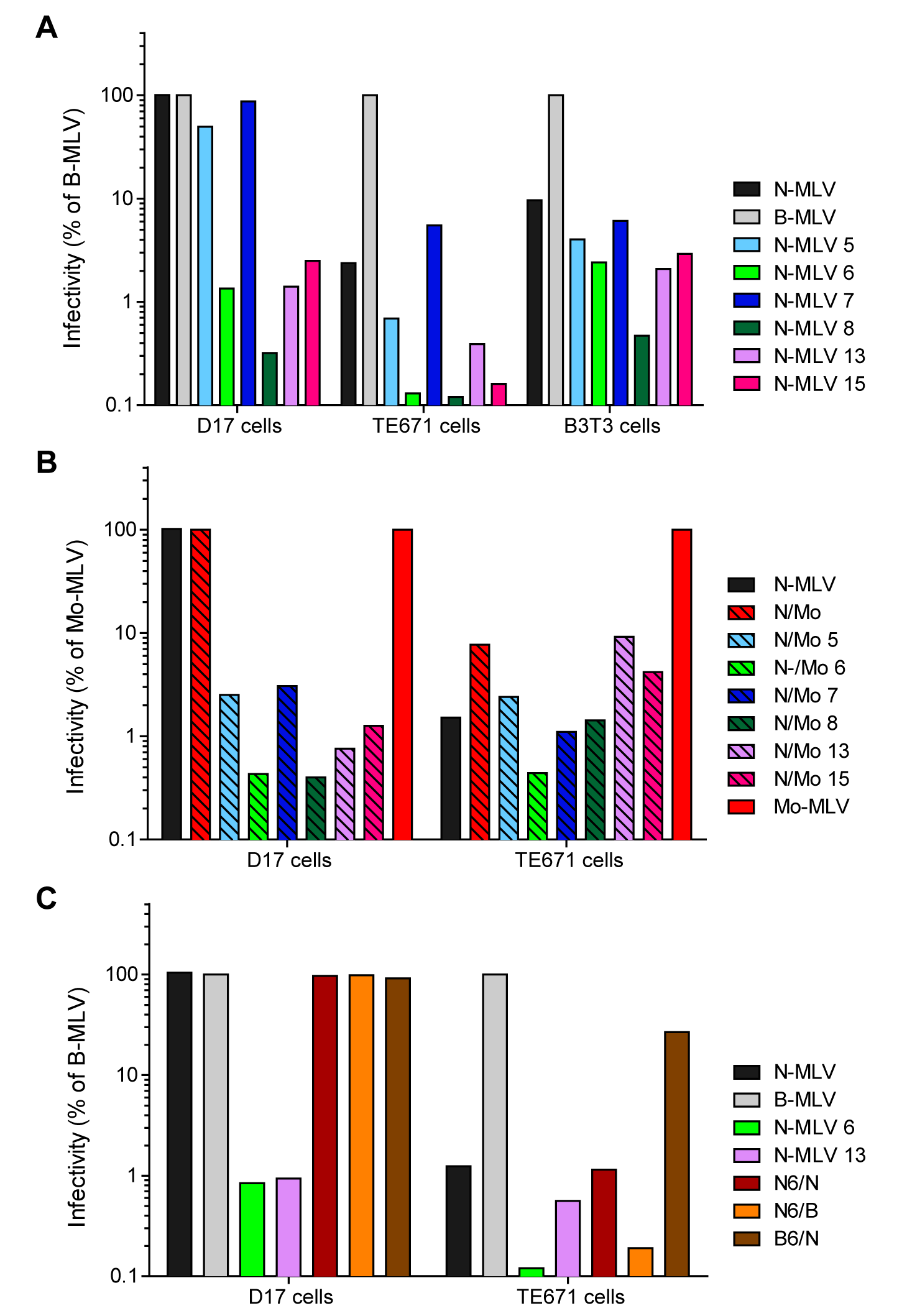

Supplement: Additional file 2 — Infectivity of particles used in TRIM5alpha and Fv1 saturation assays (Figure4). (A) LacZ-encoding N-MLV tester viruses, with or without p12 mutations, or B-MLV were synthesized in 293T cells and used in TRIM5alpha and Fv1b abrogation assays (Figure 4A and 4B). Equal RT-units of VLPs were used to challenge D17, TE671 and B3T3 cells and infectivity was measured by detection of β–galactosidase activity in a chemiluminescent reporter assay. Infectivity is plotted as a percentage of the B-MLV control. (B) The infectivity of N/Mo LacZ tester viruses that were used for TRIM5alpha abrogation assays (Figure 4C) was tested in D17 and TE671 cells as in (A). Infectivity is plotted as the percentage of the Mo-MLV control. (C) The infectivities of the mixed particles N6/B, B6/N and N6/N and the control viruses that were used for TRIM5alpha abrogation assays (Figure 4D) were tested in D17 and TE671 cells as in (A). [file 1742-4690-9-83-S2.tiff]

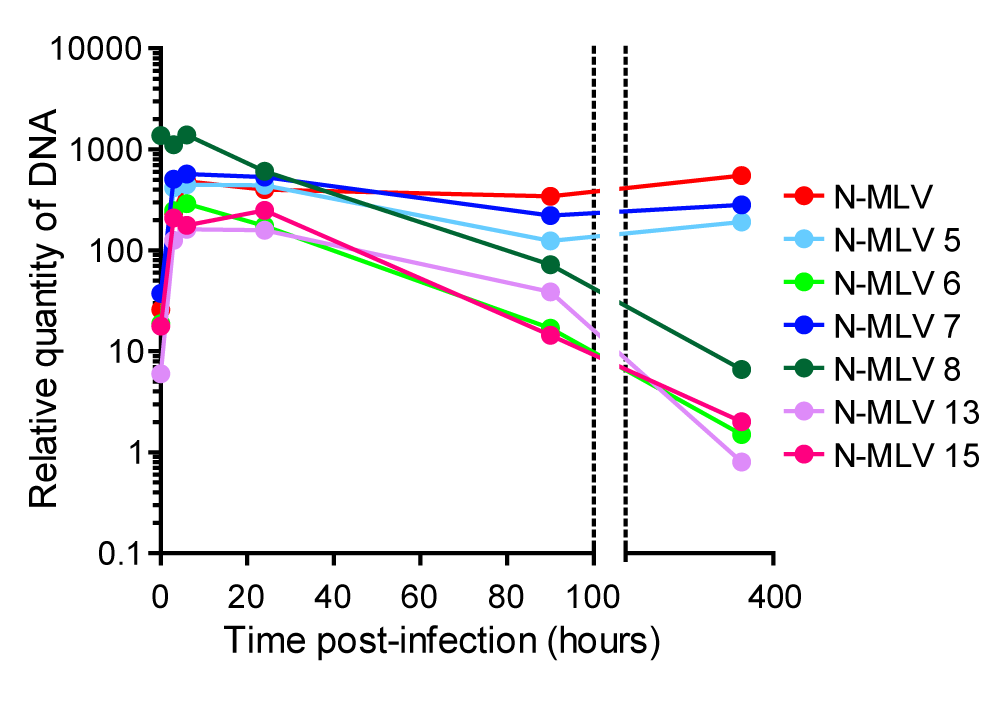

Supplement: Additional file 3 — Quantification of viral cDNA levels in D17 cells. Wild type and mutant N-MLV VLPs were produced in 293T cells by transient transfection and equal RT-units of VLPs were used to challenge D17 cells. Total DNA was isolated at various times post infection as indicated, and the relative amounts of second strand extension were measured using qPCR. Results are representative of three independent experiments. [file 1742-4690-9-83-S3.tiff]

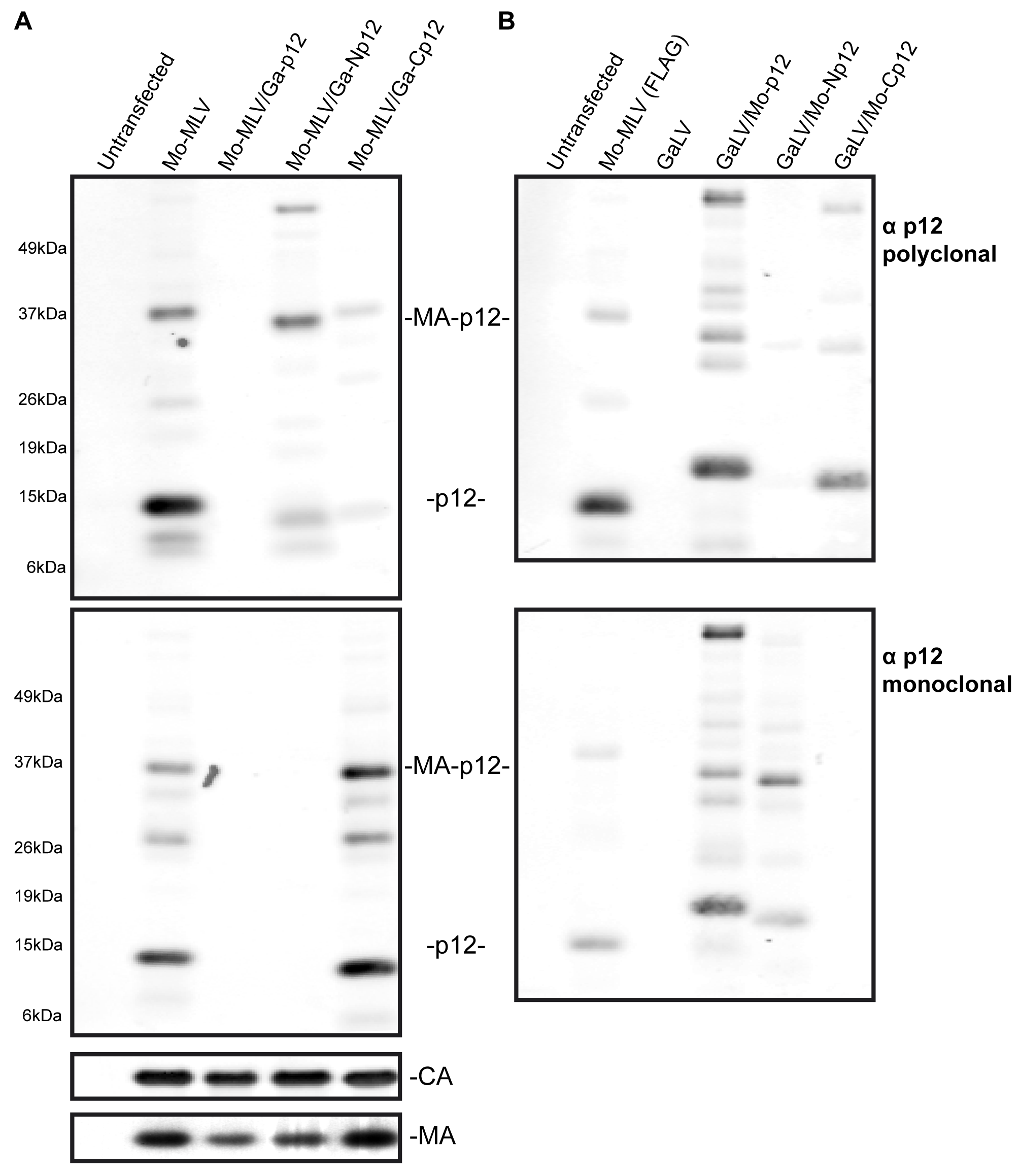

Supplement: Additional file 4 — Immunoblot analysis of Mo-MLV/GaLV chimeras. LacZ-encoding Mo-MLV/GaLV p12 chimeric VLPs (A) and GaLV/Mo-MLV p12 chimeric VLPs (B) were produced by transfection of 293T cells (as in Figure 7). Equal RT-units of particles were concentrated through 20% (w/v) sucrose cushions and lysed in SDS loading dye. Viral proteins were separated on a 10% polyacrylamide gel by SDS-PAGE and p12 was detected with an anti-MLV p12 polyclonal antibody (A and B, top panels) and an anti-MLV p12 monoclonal antibody (A, middle panel; B, bottom panel). The approximate sizes of p12 and MA-p12 are indicated. GaLV p12 is not detected with either of these antibodies. In addition, the Mo-MLV based chimeras were probed with anti-MLV CA and anti-MLV MA antibodies (A, bottom panels). These antibodies did not cross react with GaLV Gag proteins. [file 1742-4690-9-83-S4.tiff]
